# Decoding the Chloroplast Genome of Bitterwood (*Picrasma quassioides*): Structure, Variability, and Evolutionary Relationships

**DOI:** 10.1002/ece3.71245

**Published:** 2025-04-10

**Authors:** Qin Liu, Huaxi Huang, Jinhang Lin, Fanglin Liu, Xuexue Wang, Rong Chen, Xiaoshan Geng

**Affiliations:** ^1^ College of Biological Sciences and Technology Yili Normal University Yining China; ^2^ Guangxi Key Laboratory of Agricultural Resources Chemistry and Biotechnology Yulin Normal University Yulin China; ^3^ College of Agricultural Engineering Guangxi Vocational University of Agriculture Nanning China

**Keywords:** chloroplast genome, codon usage, nucleotide polymorphism, phylogenetics, *Picrasma quassioides*, selective pressure, Simaroubaceae

## Abstract

The chloroplast genome of *Picrasma quassioides* , a medicinally significant plant in traditional Asian medicine, was sequenced and analyzed to understand its genetic architecture and evolutionary relationships within Simaroubaceae. High‐throughput sequencing revealed a 160,013‐bp circular genome with a typical quadripartite structure, encoding 132 genes including 87 protein‐coding genes, 37 tRNA genes, and 8 rRNA genes. Comparative analysis with other Simaroubaceae species identified distinct patterns of nucleotide diversity between single‐copy and repeat regions, while examination of IR boundaries revealed dynamic evolutionary processes. Analysis of 101 SSR loci and 48 repeat sequences provided insights into genome organization and potential molecular markers for species identification. Selective pressure analysis across 78 protein‐coding genes demonstrated predominant purifying selection (average *K*_a_/*K*_s_ ratio 0.23), with evidence of positive selection in specific genes. Phylogenetic reconstruction using 77 protein‐coding sequences confirmed Simaroubaceae's monophyly and revealed a close evolutionary relationship with Rutaceae. These findings advance our understanding of chloroplast genome evolution within Simaroubaceae while providing molecular tools for authentication and breeding of this valuable medicinal species. The comprehensive genomic characterization establishes a foundation for investigating the genetic basis of therapeutic properties in *P. quassioides* and facilitating its conservation.

## Graphical abstract

This study reports the complete chloroplast genome of *Picrasma quassioides* , revealing its quadripartite structure, 132 genes, and insights into the evolution of Simaroubaceae. We identified potential molecular markers for species identification, found evidence of selective pressures, and established a robust phylogenetic framework, thereby providing both evolutionary understanding and tools for conservation.

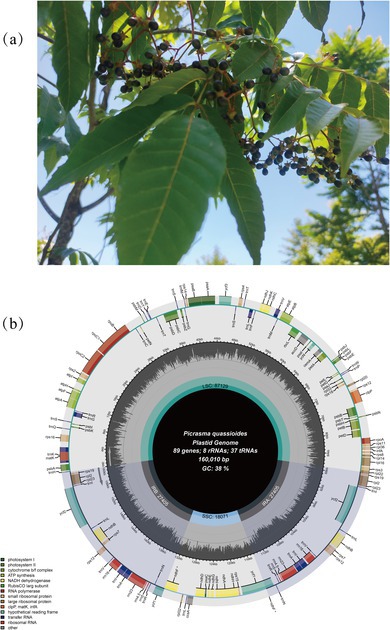

## 1 Introduction

*Picrasma quassioides* (D. Don) Benn., a deciduous tree of the Simaroubaceae family, is a pharmacologically significant species widely utilized in traditional Asian medicinal systems for its heat‐clearing and detoxification properties (Zhao et al. 2019). Officially documented in the *Chinese Pharmacopeia* (2020 edition), its bitter taste (*ku*) and cold nature (*han*) underpin therapeutic applications against wind‐heat induced ailments, including respiratory infections, inflammatory pharyngitis, bacillary dysentery, and chronic eczema (Commission, C.P. 2020). Phytochemical investigations have identified over 200 bioactive constituents spanning eight major classes: quassinoids (e.g., nigakilactones), indole alkaloids (e.g., cantleyine derivatives), triterpenoids, volatile terpenes, flavonoid glycosides, steroidal saponins, coumarins, and phenolic derivatives (Scragg and Allan 1993; Huang et al. 2013; Xu et al. 2016; Mohd Jamil et al. 2020). Contemporary pharmacological research has validated multiple bioactivities, including anti‐cancer, anti‐inflammatory, antibacterial, antihypertensive, antipyretic, antivenom, and antimalarial effects (Song et al. 2014; Lee et al. 2016; Xie et al. 2020; Mohd Jamil et al. 2020; Revanasiddappa et al. 2024; So et al. 2024). Despite escalating demand for its medicinal extracts, wild populations are critically threatened by unsustainable harvesting, while genomic resources and molecular mechanisms underlying its biosynthetic pathways remain poorly characterized (Qin et al. 2022), necessitating urgent conservation and biotechnological interventions.

The conservation and sustainable cultivation of *P. quassioides* require advanced genomic insights, particularly through chloroplast genome analysis. As semi‐autonomous organelles governing photosynthesis and secondary metabolite synthesis (Daniell et al. 2016), chloroplasts possess a conserved circular double‐stranded DNA structure comprising four distinct regions: a large single‐copy (LSC, ~85 kb), a small single‐copy (SSC, ~18 kb), and two inverted repeat (IR, ~25 kb) regions, with total sizes typically ranging from 120 to 160 kb across angiosperms (Bock 2015). The utility of chloroplast DNA (cpDNA) in evolutionary studies stems from its uniparental inheritance, low nucleotide substitution rates, and highly conserved gene order—attributes enabling robust phylogenetic reconstructions and species delineation (Clegg et al. 1994; Scarcelli et al. 2011; Dong et al. 2013). Recent advancements in next‐generation sequencing (NGS) platforms have revolutionized cpDNA research, facilitating rapid assembly of plastomes and comparative analyses at unprecedented resolutions (Scarcelli et al. 2024; Yao et al. 2025). Nevertheless, within the ecologically significant yet genomically understudied Simaroubaceae family (Pirani et al. 2021), *P. quassioides* remains devoid of comprehensive chloroplast genomic resources, hindering both phylogenetic classification and conservation genomics.

To address these knowledge gaps, we present the complete chloroplast genome sequence of *P. quassioides* , obtained through Illumina high‐throughput sequencing technology. Our comprehensive analysis encompasses structural features (including gene content, repeats, and codon usage bias), comparative assessment of IR region variation and nucleotide diversity within Simaroubaceae, and phylogenetic reconstruction. These analyses not only aim to resolve taxonomic ambiguities within the family but also provide molecular markers for germplasm authentication and inform strategies for in situ conservation of this pharmacologically vital species.

## 2 Materials and Methods

### 2.1 Plant Material, DNA Extraction, and Sequencing

Fresh leaves of *P. quassioides* were collected from mature individuals (*n* = 5) in the karst terrain of Guangxi Zhuang Autonomous Region, China (23°06′ N, 107°03′ E; elevation 450 m). No specific permits were required for the sample collection as the study did not involve endangered or protected species and the sampling location was not in a protected area. Voucher specimens (HZ2022‐05) were authenticated by Dr. Cansheng Zhu and deposited at the Guangxi Academy of Forestry Sciences Herbarium (contact: gxns1803@163.com). Genomic DNA was extracted from silica‐dried leaves using a modified CTAB protocol with polyvinylpyrrolidone (PVP‐40) inclusion for polyphenol removal (Yang et al. 2014), followed by RNase A (Thermo Scientific) treatment. DNA integrity was verified through 1% agarose gel electrophoresis (120 V, 45 min), and purity quantified via NanoDrop OneC spectrophotometer (A260/A280 = 1.82 ± 0.03; A260/A230 = 2.15 ± 0.05).

Whole‐genome sequencing was performed on an Illumina NovaSeq 6000 platform (Illumina, San Diego, CA, USA) at Huitong Biotechnology Co. Ltd. (Shenzhen, China), generating 150 bp paired‐end libraries with 350 bp insert sizes. Initial sequencing produced 64.12 million raw reads (9.62 Gb total data), which were quality‐filtered using Trimmomatic v0.33 (Bolger et al. 2014) with parameters: SLIDINGWINDOW:4:20, MINLEN:50. This process retained 59.64 million clean reads for subsequent analysis.

### 2.2 Chloroplast Genome Assembly and Annotation

Chloroplast genomes were assembled independently for all five individuals using GetOrganelle v1.7.1 (Jin et al. 2020), with default *k*‐mer parameters (*k* = 21, 45, 65, 85, 105). The resulting assemblies, each 160,013 bp, were aligned using MAFFT v7.487 (Katoh and Standley 2013) to assess intra‐specific variation, revealing no nucleotide differences among them. Consequently, a single representative sequence (from specimen HZ2022‐05‐01) was selected for detailed analysis. Genome annotation was performed using the CPGAVAS2 platform (Liu et al. 2012), with the *Ailanthus altissima* chloroplast genome (GenBank: NC_037696.1) as reference. Annotations were manually refined using Geneious Prime v2023.2.1 (Kearse et al. 2012). The structural organization of the annotated genome was visualized using Chloroplot (Zheng et al. 2020) with GC skew adjustment. The complete chloroplast genome sequence was deposited in GenBank (accession: NC_067857.1).

### 2.3 Analysis of Relative Codon Usage and Repetitive Sequence

Relative synonymous codon usage (RSCU) values for 87 protein‐coding genes (PCGs) were calculated using CodonW v1.4.2 (Sharp et al. 1986). REPuter v3.0 (Kurtz 2001) identified four repeat types with parameters: Hamming distance = 3, minimum repeat size = 30 bp, and *E*‐value ≤ 1e‐5. Simple sequence repeats (SSRs) were detected via MISA v2.1 (Beier et al. 2017), with minimum repeat thresholds of 10 for mononucleotides, 5 for dinucleotides, 4 for trinucleotides, and 3 for tetra‐, penta‐, and hexanucleotides.

### 2.4 Comparative Analysis of Chloroplast Genomes

Inverted repeat (IR) region dynamics were analyzed among seven Simaroubaceae species: *P. quassioides* (NC_067857.1), *Eurycoma longifolia* (MH751519.1), *A. altissima* (NC_037696.1), *Leitneria floridana* (NC_030482.1), *Simarouba versicolor* (NC_088060.1), *Simarouba amara* (NC_085162.1), and *Brucea javanica* (NC_063730.1), using IRscope (Amiryousefi et al. 2018). Nucleotide diversity (*π*) was assessed using DnaSP v6 (Rozas et al. 2017), employing a 600 bp sliding window with 200 bp steps to evaluate chloroplast genome variability.

### 2.5 Analysis of Selective Pressure

Selective pressure analysis was performed on shared protein‐coding genes among the seven Simaroubaceae species using KaKs_calculator v3.0 (Zhang 2022). Synonymous (*K*_s_) and non‐synonymous (*K*_a_) substitution rates were calculated through pairwise comparisons. Interpretation criteria followed standard thresholds: *K*_a_/*K*_s_ > 1 indicated positive selection, *K*_a_/*K*_s_ = 1 suggested neutral selection, and *K*_a_/*K*_s_ < 1 reflected purifying selection.

### 2.6 Phylogenetic Analysis

To determine the phylogenetic position of *P. quassioides* within Sapindales, we analyzed 41 complete chloroplast genomes (including all seven Simaroubaceae species) with *Panax ginseng* and *P. notoginseng* (Araliaceae) as outgroups. Seventy‐seven shared protein‐coding genes were aligned using MAFFT v7.487 (Katoh and Standley 2013). Poorly aligned regions were removed using Gblocks v0.91b (Talavera and Castresana 2007). Phylogenetic reconstruction employed maximum likelihood methods in IQ‐TREE v1.6.12 (Minh et al. 2020), using the GTR + F + R3 substitution model selected by ModelFinder (Kalyaanamoorthy et al. 2017). Tree robustness was assessed with 5000 ultrafast bootstrap replicates, and the final tree was visualized using the Chiplot online tool (https://www.chiplot.online/).

## 3 Results

### 3.1 Structural Characterization of the *P. quassioides* Chloroplast Genome

**FIGURE 1:**
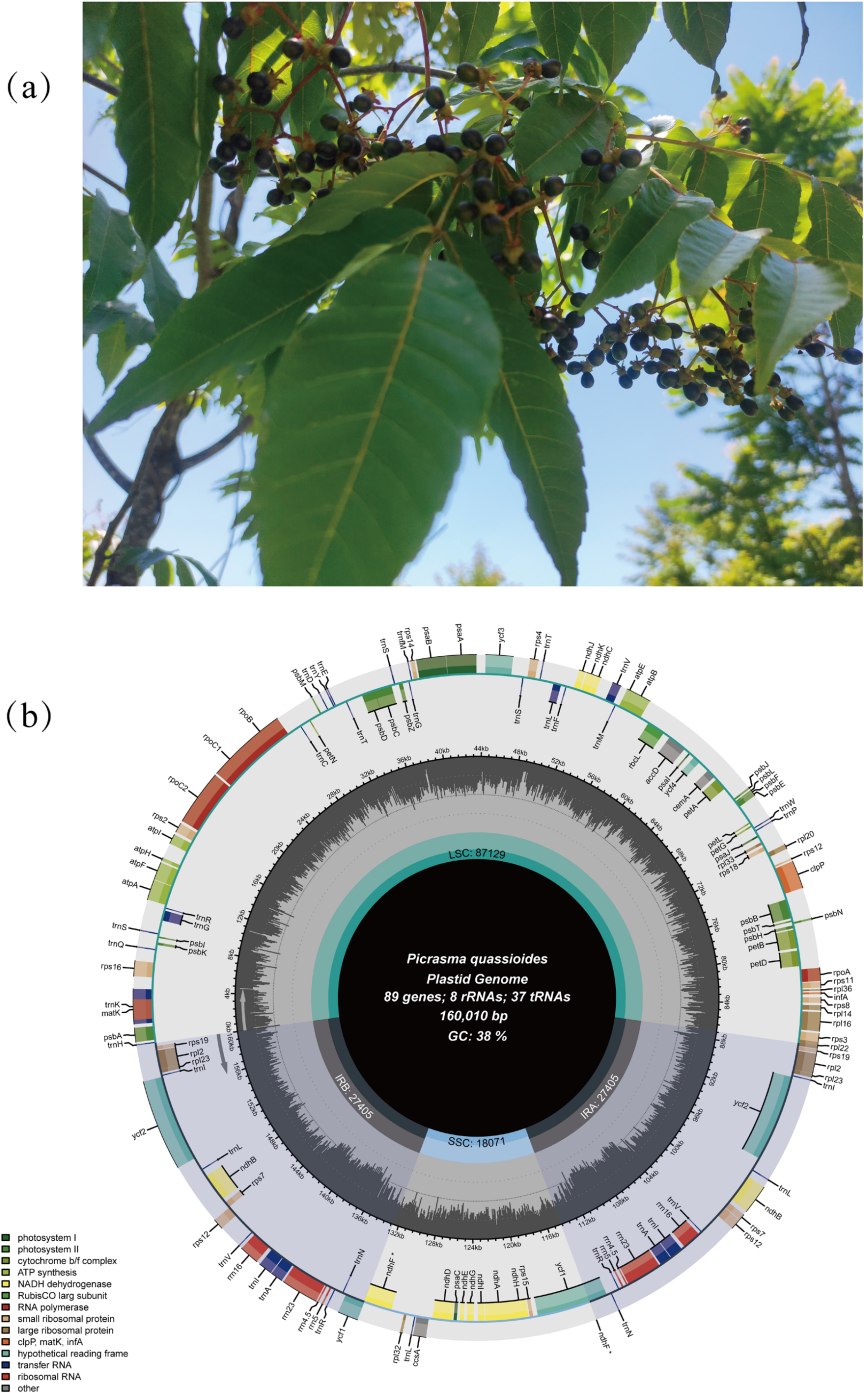
Morphology and chloroplast genome map of *Picrasma quassioides* (a) Photograph of *P. quassioides* leaves and fruits (b) circular map of the *P. quassioides* chloroplast genome. The outermost circle displays the gene map, with genes color‐coded based on their functional categories. The inner circle depicts the genome regions: Large single copy (LSC), small single copy (SSC), and two inverted repeat regions (IRA and IRB). The gray inner circles represent GC content across the genome. The total size and GC content of the genome are shown at the center of the map.

Figure 1a presents the morphology of *P. quassioides* , showcasing its leaves and fruits. The complete chloroplast genome of *P. quassioides* was assembled and characterized as a circular DNA molecule with a total length of 160,013 bp (Figure 1b). It exhibits the conserved quadripartite structure typical of angiosperm chloroplast genomes. This structure comprises a large single‐copy (LSC) region (87,129 bp, 54.45%), a small single‐copy (SSC) region (18,072 bp, 11.29%), and two inverted repeat (IR) regions (27,406 bp each, totaling 34.26%).

Analysis of regional nucleotide composition demonstrated significant structural heterogeneity (Table 1). The overall GC content was 37.97%, masking substantial intragenomic variation. Specifically, IR regions exhibited the highest GC content (42.67%), primarily due to the enrichment of rRNA gene clusters. In contrast, the SSC region displayed the lowest GC content (32.41%). Within protein‐coding sequences (CDS), GC content showed codon position‐dependent stratification. The first codon position maintained the highest GC content (45.76%), while the third position exhibited the lowest (31.17%). This pattern is consistent with selective pressures favoring AT‐rich synonymous codons at translationally less constrained sites.

**TABLE 1:** Nucleotide composition in different regions of the chloroplast genome of *Picrasma quassioides* .

Region	Size (bp)	A (%)	T (%)	G (%)	C (%)	GC (%)
Total genome	160,013	30.63	31.4	18.77	19.2	37.97
LSC	87,129	31.16	32.68	17.56	18.6	36.16
IRA	27,406	28.82	28.51	22.11	20.56	42.67
SSC	18,072	34.02	33.57	16.81	15.6	32.41
IRB	27,406	28.51	28.82	20.56	22.11	42.67
CDS	80,997	30.58	31.08	20.5	17.85	38.35
1st codon position	26,999	30.65	23.59	26.96	18.8	45.76
2nd codon position	26,999	29.43	32.46	17.95	20.17	38.12
3rd codon position	26,999	31.65	37.19	16.59	14.58	31.17

Genome annotation identified 132 functional genes, including 87 protein‐coding genes, 37 transfer RNA (tRNA) genes, and 8 ribosomal RNA (rRNA) genes (Table 2). Further analysis of gene structure revealed complex intron patterns. Sixteen genes were found to contain single introns, including *ndhA*, *ndhB*, *petB*, *petD*, *atpF*, *rpl16*, *rpl2*, *rps12*, *rps16*, *rpoC1*, *trnA‐UGC*, *trnG‐UCC*, *trnI‐GAU*, *trnL‐UAA*, *trnK‐UUU*, and *trnV‐UAC*. Two genes (*clpP* and *ycf3*) contained double introns. The remaining genes lacked intronic sequences entirely, suggesting phylogenetic divergence in splicing requirements and functional constraints among chloroplast genes.

**TABLE 2:** Gene content and organization in the *Picrasma quassioides* chloroplast genome.

Functional category	Gene group	Gene name
Photosynthesis	Subunits of photosystem I	*psaA*, *psaB*, *psaC*, *psaI*, *psaJ*
	Subunits of photosystem II	*psbA*, *psbB*, *psbC*, *psbD*, *psbE*, *psbF*, *psbH*, *psbI*, *psbJ*, *psbK*, *psbL*, *psbM*, *psbN*, *psbT*, *psbZ*
	Subunits of NADH dehydrogenase	ndhA**, ndhB**(×2), *ndhC*, *ndhD*, *ndhE*, *ndhF*, *ndhG*, *ndhH*, *ndhI*, *ndhJ*, *ndhK*
	Subunits of cytochrome b/f complex	*petA*, petB**, petD**, *petG*, *petL*, *petN*
	Subunits of ATP synthase	*atpA*, *atpB*, *atpE*, atpF**, *atpH*, *atpI*
	Large subunit of RuBisCO	*rbcL*
Self‐replication	Proteins of large ribosomal subunit	*rpl14*, rpl16**, rpl2**(×2), *rpl20*, *rpl22*, rpl23**(×2), *rpl32*, *rpl33*, *rpl36*
	Proteins of small ribosomal subunit	*rps11*, rps12**(×2), *rps14*, *rps15*, rps16**, *rps18*, rps19**(×2), *rps2*, *rps3*, *rps4*, rps7**(×2), *rps8*
	Subunits of RNA polymerase	*rpoA*, *rpoB*, rpoC1**, *rpoC2*
	Ribosomal RNAs	rrn16**(×2), rrn23**(×2), *rrn4.5*(×2), *rrn5*(×2)
	Transfer RNAs	trnA‐UGC**(×2), *trnC‐GCA*, *trnD‐GUC*, *trnE‐UUC*, *trnF‐GAA*, *trnG‐GCC*, trnG‐UCC**, *trnH‐GUG*, trnI‐CAU**(×2), trnI‐GAU**(×2), trnK‐UUU**,* trnL‐CAA**(×2), trnL‐UAA**, *trnL‐UAG*, *trnM‐CAU*, trnM‐GUU**(×2), *trnP‐UGG*, *trnQ‐UUG*, trnR‐ACG**(×2), *trnR‐UCU*, *trnS‐GCU*, *trnS‐GGA*, *trnS‐UGA*, *trnT‐GGU*, *trnT‐UGU*, *trnV‐GAC*(×2), *trnV‐UAC*, *trnW‐CCA*, *trnY‐GUA*, *trnM‐CAU*
Other genes	Maturase	*matK*
	Protease	clpP**
	Envelope membrane protein	*cemA*
	Acetyl‐CoA carboxylase	*accD*
	c‐type cytochrome synthesis	*ccsA*
	Translation initiation factor	*infA*
Unknown	Conserved hypothetical chloroplast ORF	*ycf1*(×2), *ycf2*(×2), ycf3**, *ycf4*

*Note:* Gene*: Gene with one intron; Gene**: Gene with two introns; Gene(2): Number of copies of multi‐copy genes.

### 3.2 Analysis of Repetitive Elements and Simple Sequence Repeats

**FIGURE 2:**
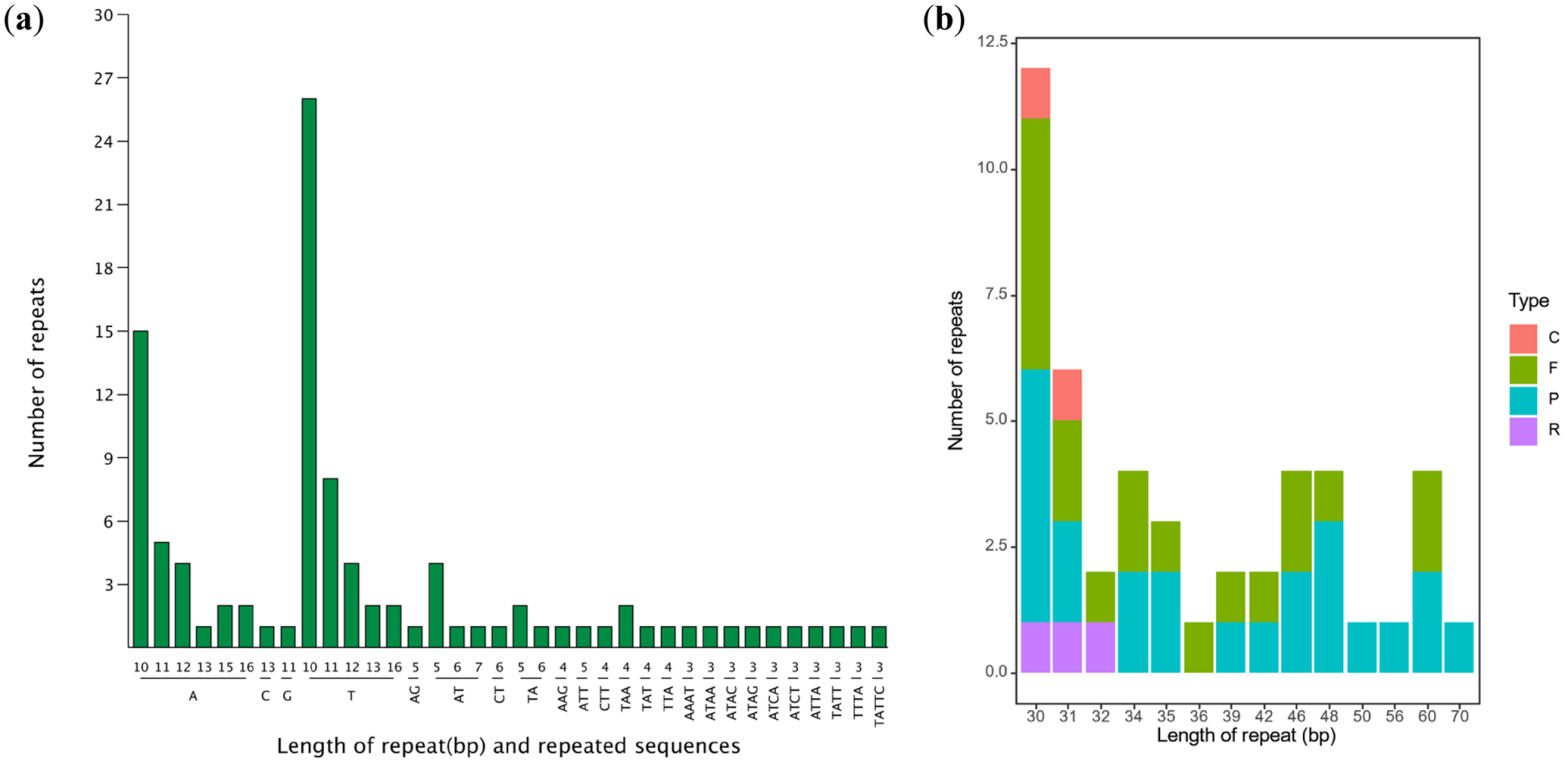
Distribution and characterization of repetitive elements in the *Picrasma quassioides* chloroplast genome: (a) Frequency distribution of simple sequence repeats (SSRs) by repeat type and repeat unit size, and (b) quantitative analysis of long repeat sequences, categorized by repeat type (C, complementary; F, forward; P, palindromic; R, reverse) and length distribution.

Analysis of the *P. quassioides* chloroplast genome revealed the presence of 101 simple sequence repeats (SSRs), exhibiting distinct compositional and length characteristics (Figure 2a). Mononucleotide repeats, predominantly poly‐A/T repeats, were the most abundant SSR type (72.28%, 73/101), with repeat units ranging from 10 to 16. Dinucleotide SSRs accounted for 10.89% (11/101) and were primarily composed of AT/TA motifs (9 loci) with 5–7 repeat units. Tri‐ to hexanucleotide repeats represented 16.83% (17/101) of the SSRs, displaying 3–5 repeat units. The predominance of A/T‐rich SSRs aligns with the genome's overall AT bias (62.03%) and suggests their utility as molecular markers for population genetics and species authentication, particularly valuable for conserving this medicinally significant species.

Comprehensive repeat sequence analysis identified 47 distinct repeat elements, categorized into four types: forward (F), reverse (R), complementary (C), and palindromic (P) repeats (Figure 2b). The majority of these repeats ranged in length from 30 to 60 base pairs. Palindromic repeats were the most frequent type, with 23 occurrences. Forward repeats were the second most common type, observed 19 times. Reverse and complementary repeats were less frequent and typically ranged from 30 to 32 base pairs in length.

### 3.3 Patterns of Codon Usage Bias in Protein‐Coding Genes

**FIGURE 3:**
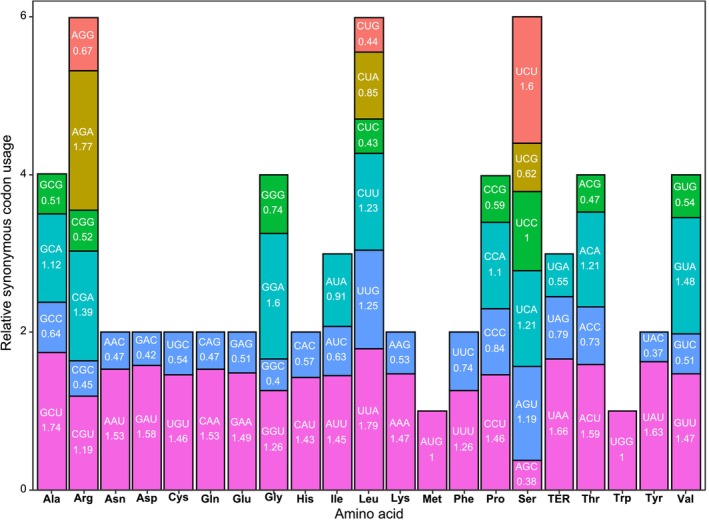
Codon usage bias analysis in the *Picrasma quassioides* chloroplast genome. The relative synonymous codon usage (RSCU) value for each codon is shown, indicating the observed frequency of a codon relative to the expected frequency under the assumption of equal usage among synonymous codons. RSCU values greater than 1 indicate preferential usage of a codon, while values less than 1 indicate less frequent usage than expected based on equal synonymous codon frequencies.

Codon usage analysis revealed significant non‐random preferences in the *P. quassioides* chloroplast genome, as evidenced by relative synonymous codon usage (RSCU) values (Figure 3). Notable codon usage biases were observed for several amino acids. For instance, arginine codons AGA and CGU exhibited markedly preferential usage (RSCU values of 1.77 and 1.19, respectively) compared to their synonymous counterparts AGG and CGG, which showed considerably lower usage (RSCU values of 0.67 and 0.52, respectively). A similar pattern was evident for glycine, where GGA displayed a significantly higher RSCU value (1.6) than GGC (RSCU 0.4). Likewise, leucine codons UUA and CUC showed elevated usage frequencies (RSCU values of 1.79 and 1.23, respectively), in contrast to the less frequent codon CUG (RSCU 0.44). Among stop codons, UAA emerged as the predominantly utilized termination codon. These systematic variations in codon usage patterns suggest that specific selective pressures are shaping the protein‐coding regions of the chloroplast genome. These biases likely reflect coevolution with tRNA availability, enhancing translational efficiency in chloroplast genes essential for photosynthesis and organelle function, which indirectly supports the plant's physiological processes (Parvathy et al. 2022).

### 3.4 Comparative Analysis of IR Region Boundaries

**FIGURE 4:**
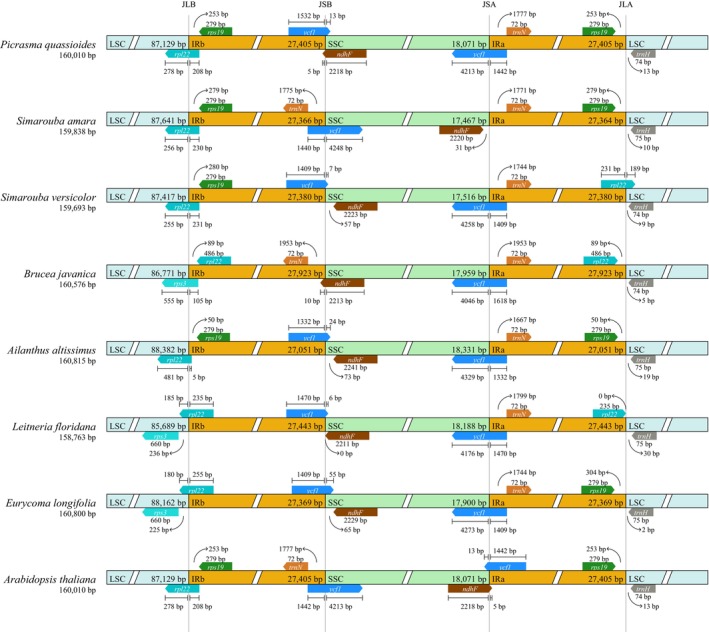
Structural comparison of inverted repeat (IR) boundaries across seven Simaroubaceae chloroplast genomes. The diagram illustrates four key junction points: LSC/IRb (JLB), IRb/SSC (JSB), SSC/IRa (JSA), and IRa/LSC (JLA). Numbers within boxes indicate the distance (in base pairs) between gene terminals and boundary positions. Arrows demonstrate the relative positions and orientations of genes spanning these boundaries.

Comparative examination of inverted repeat (IR) junction boundaries across seven Simaroubaceae chloroplast genomes revealed conserved evolutionary patterns with species‐specific structural modifications (Figure 4). The complete chloroplast genomes exhibited limited size variation (158,736 bp in *L. floridana* to 160,815 bp in *A. altissima* ), maintaining conserved quadripartite architecture despite boundary position variations. The JLB junction (LSC/IRb boundary) demonstrated substantial interspecific divergence, predominantly located within the *rpl22* coding region but exhibiting variable protrusion into IRb (5 bp in *A. altissima* vs. 255 bp in *E. longifolia*). Notably, *B. javanica* diverged from this pattern by positioning the JLB within *rps3*, with *rpl22* located 89 bp upstream of the boundary interface.

Conservation patterns emerged at the JSB junction (SSC/IRb boundary), consistently intersecting the pseudogenized *ycf1* sequence across all taxa, though with moderate variation in SSC penetration depth (6–55 bp). In contrast, the JSA junction (SSC/IRa boundary) displayed greater plasticity, showing functional *ycf1* extensions into IRa ranging from 1168 bp to 1332 bp. The JLA junction (IRa/LSC boundary) exhibited three structural configurations: Cluster I species ( *P. quassioides* , *E. longifolia*, *A. altissima* ) positioned the boundary between *rps19* and *trnH*; Cluster II species contained the junction within *rpl22* with LSC extensions (0–189 bp); while *B. javanica* (Cluster III) uniquely located the boundary in the *rpl22‐trnH* intergenic spacer, maintaining 89 bp between *rpl22* and the junction—a feature absent in other examined taxa. These boundary variations suggest differential evolutionary constraints acting on IR expansion/contraction processes within Simaroubaceae.

### 3.5 Pattern Analysis of Nucleotide Diversity

**FIGURE 5:**
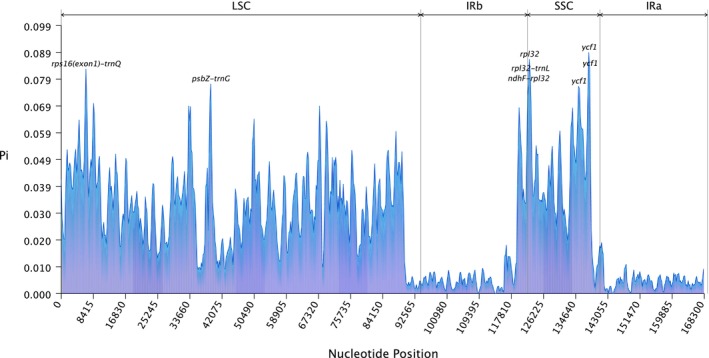
Nucleotide diversity analysis across seven Simaroubaceae chloroplast genomes. The graph displays nucleotide diversity (*π*) values calculated using a sliding window approach. Peaks indicate regions of high sequence variability, while troughs represent conserved regions. Notable peaks correspond to specific genic and intergenic regions. The *x*‐axis represents the position along the aligned sequences, and the *y*‐axis shows the nucleotide diversity values.

A comparative analysis of nucleotide diversity across seven Simaroubaceae chloroplast genomes revealed distinct patterns of sequence variation (Figure 5). Multiple sequence alignment and subsequent nucleotide polymorphism analysis demonstrated regional heterogeneity in sequence conservation, with clear differences between single‐copy and repeat regions.

The genome‐wide nucleotide diversity (*π*) ranged from 0 to 0.089, with a mean value of 0.025. Structurally, the large single‐copy (LSC) and small single‐copy (SSC) regions exhibited substantially higher sequence variability compared to the more conserved inverted repeat (IR) regions. Several genomic hotspots of elevated nucleotide diversity (*π* > 0.075) were identified, including both genic and intergenic regions. These hotspots included the *ycf1* gene region, the *rpl32* gene region, and several intergenic spacers (*rps16* exon1‐*trnQ*, *rpl32‐trnL*, *ndhF‐rpl32*, and *psbZ‐trnG*). The *ycf1* region within the SSC was particularly noteworthy, demonstrating multiple sites of high sequence variability. This finding suggests its potential utility as a molecular marker for evolutionary studies and species discrimination within Simaroubaceae.

### 3.6 Analysis of Selective Evolutionary Pressure in Simaroubaceae Chloroplast Genes

**FIGURE 6:**
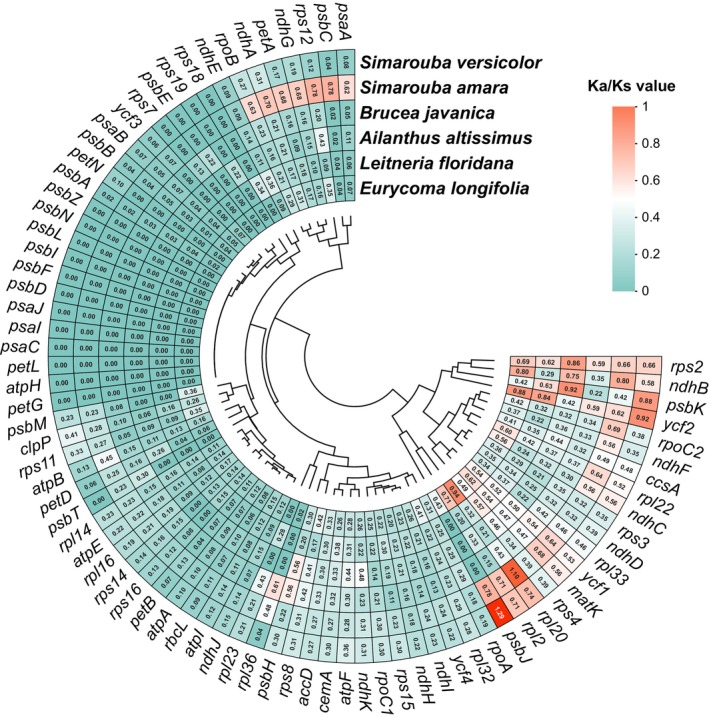
Distribution of selective pressure across chloroplast genes in Simaroubaceae. The graph presents *K*_a_/*K*_s_ ratios for 78 protein‐coding genes shared between *Picrasma quassioides* and six other Simaroubaceae species.

A comparative analysis of selective pressure among seven Simaroubaceae species revealed distinct patterns of evolutionary constraint across chloroplast protein‐coding genes (Figure 6). Using the *P. quassioides* chloroplast genome as a reference, the ratio of non‐synonymous (*K*_a_) to synonymous (*K*_s_) substitution rates was examined across 78 shared protein‐coding genes.

The analysis yielded an average *K*_a_/*K*_s_ ratio of 0.23 across all examined genes, indicating a predominant pattern of purifying selection throughout the chloroplast genome. This observation suggests strong evolutionary constraints maintaining functional conservation of most chloroplast genes. However, specific genes exhibiting divergent evolutionary patterns were also identified. Most notably, the *psbJ* gene in *E. longifolia* and the *rpl20* gene in *L. floridana* demonstrated *K*_a_/*K*_s_ ratios exceeding 1.0, suggesting these genes have undergone positive selection. The remaining genes consistently showed *K*_a_/*K*_s_ ratios below 1.0, confirming widespread purifying selection as the dominant evolutionary force in Simaroubaceae chloroplast genomes. This pattern of selective pressure suggests that most chloroplast genes maintain essential functions that are highly conserved across species, while allowing for occasional adaptive evolution in specific genes.

### 3.7 Phylogenetic Analysis and Evolutionary Relationships

**FIGURE 7:**
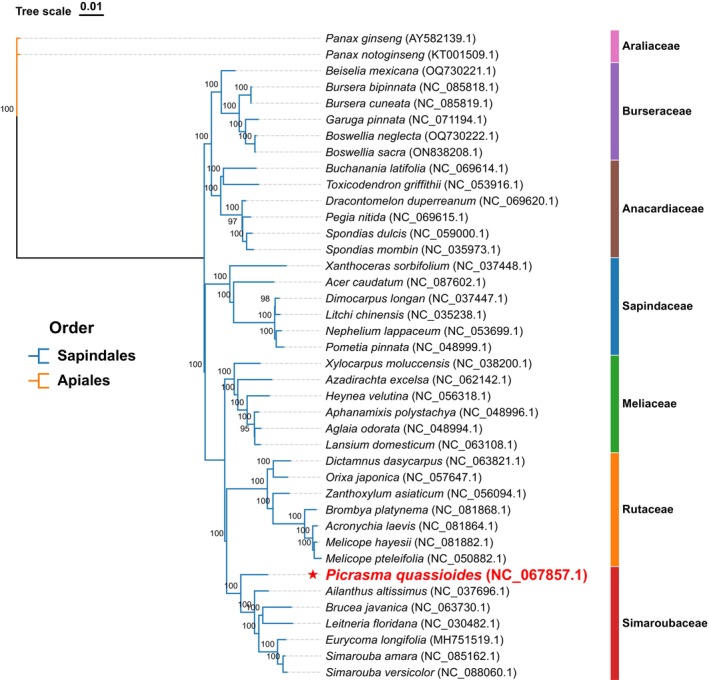
Molecular phylogeny of Sapindales based on chloroplast protein‐coding genes. The maximum likelihood tree was constructed using 77 protein‐coding sequences from 41 species, including 39 Sapindales and 2 Araliaceae outgroups. Numbers at nodes indicate bootstrap support values from 5000 replicates. *Picrasma quassioides* (marked with a red star) is shown in the context of major Sapindales lineages.

A comprehensive phylogenetic analysis was conducted to elucidate the evolutionary relationships within Sapindales, utilizing 77 shared protein‐coding genes from chloroplast genomes across 41 species (Figure 7). The analysis included 39 Sapindales species and two Araliaceae species ( *P. ginseng* and *Panax notoginseng*) as phylogenetic outgroups. The resulting Maximum Likelihood (ML) tree revealed well‐resolved evolutionary relationships, supported by robust bootstrap values exceeding 90% across all major branches.

The phylogenetic reconstruction demonstrated clear ordinal‐level differentiation between Sapindales and Apiales, with the tree topology resolving into two major clades and five distinct subclades. Each subclade received maximal bootstrap support (100%), indicating high confidence in the recovered family‐level relationships. Within this framework, the analysis revealed several significant evolutionary patterns at different taxonomic levels.

At the family level, *P. quassioides* was firmly positioned within the Simaroubaceae clade, exhibiting close evolutionary relationships with *A. altissima* , *B. javanica*, *L. floridana* , *E. longifolia*, *S. amara*, and *S. versicolor*. Furthermore, the analysis revealed a particularly close evolutionary relationship between Simaroubaceae and Rutaceae, supported by a maximum bootstrap value of 100%. This sister‐group relationship was more strongly supported than relationships with other Sapindales families, including Burseraceae, Anacardiaceae, and Meliaceae.

## 4 Discussion

Sequencing the *P. quassioides* chloroplast genome offers vital insights into the genetic architecture of this important medicinal species. This research directly fills a significant knowledge void in Simaroubaceae genomics, which notably lacks detailed chloroplast genome studies, especially for medicinal plants such as *P. quassioides* . Although *P. quassioides* is traditionally used in Asian medicine for heat‐clearing and anti‐inflammatory effects (Mohd Jamil et al. 2020; Zhao et al. 2024), its organelle genome has been largely unexamined. Our in‐depth chloroplast genome analysis confirms the broad evolutionary conservation of plastid genomes within Simaroubaceae, while also identifying key genomic traits—repetitive elements, codon usage patterns, and IR boundary dynamics—that clarify plastid evolution and provide crucial resources for molecular breeding and informed *P. quassioides* conservation. The following discussion will expand on these findings, exploring their relevance to plastid genome evolution, the genetic basis of medicinal properties, and sustainable management of *P. quassioides* populations.

The complete chloroplast genome of *P. quassioides* , characterized in this study as a circular molecule of 160,013 bp, provides essential insights into the genetic architecture of this medicinally significant species. This genome exhibits the conserved quadripartite structure typical of angiosperms, comprising a large single‐copy (LSC) region (87,129 bp), a small single‐copy (SSC) region (18,072 bp), and two inverted repeat (IR) regions (27,406 bp each) (Figure 1). This structural organization and the overall genome size are consistent with those observed in other Simaroubaceae species (Saina et al. 2018; Ng et al. 2019; Scarcelli et al. 2024), indicating a stable evolutionary trajectory within this family. The regional heterogeneity in nucleotide composition, with higher GC content in the IR regions (42.67%) compared to the LSC (36.16%) and SSC (32.41%) (Table 1), is also a conserved feature, likely due to the enrichment of rRNA gene clusters in the IR, as shown by previous studies of land plant chloroplasts (Wang and Hickey 2002; Xue et al. 2019; Wang et al. 2023; Yang et al. 2023; Wu et al. 2024). The stratification of GC content across codon positions (45.76% in the first, 38.12% in the second, and 31.17% in the third) suggests selection pressures favoring AT‐rich synonymous codons at translationally less constrained sites (Liu et al. 2021; Parvathy et al. 2022).

Our analysis of repetitive elements in the *P. quassioides* chloroplast genome (Figure 2) revealed 101 SSR loci, with mononucleotide repeats (primarily A/T) being the most abundant (72.28%) and ranging from 10 to 16 bp (Figure 2a). These A/T‐rich SSRs mirror the genome's overall AT bias and have practical implications for molecular marker development. In addition, we discovered 47 larger repeats (Figure 2b). These repeats, particularly palindromic and forward types, may contribute to genome stability and are linked to genomic rearrangements that facilitate species diversification (Saina et al. 2018; Zhu et al. 2023; Kwon et al. 2023; Almeida‐Silva et al. 2024). The 101 SSR loci, particularly the abundant A/T mononucleotide repeats, offer practical tools for germplasm authentication and tracking genetic diversity in *P. quassioides* , supporting conservation efforts for this threatened medicinal species. Similarly, codon usage analysis revealed biases, such as preferential use of AGA (RSCU: 1.77) for arginine and UUA (RSCU: 1.79) for leucine (Figure 3), likely enhance translational efficiency in chloroplast genes, potentially contributing to plant fitness and adaptation under selective pressures (Parvathy et al. 2022; Robbins and Kelly 2023). The observed prevalence of the UAA stop codon highlights conserved translational termination mechanisms (Shen et al. 2024). These findings suggest future research into how such genomic features influence *P. quassioides* ' ecological and medicinal traits.

Our comparative analysis of IR junction boundaries across seven Simaroubaceae chloroplast genomes further highlighted the dynamic nature of these regions (Figure 4). While the JLB boundary generally intersected the *rpl22* gene, its variable extension into the IRb region (5–255 bp) showed a clear trend of ongoing micro‐evolutionary adjustment among species, particularly at the 5′ end of the *rpl22* sequence. The unique position of the JLB within the *rps3* gene in *B. javanica* suggests a recent evolutionary event specific to this species. The conservation of the JSB boundary at the *ycf1* pseudogene and the plasticity of the JSA boundary regarding *ycf1* extension into the IRa region demonstrate varied evolutionary constraints acting upon these loci (Wicke et al. 2011).

The patterns of nucleotide diversity also supported a regional difference of evolutionary rate (Figure 5). In line with studies on other angiosperms, the LSC and SSC regions exhibited higher sequence variability than the IR regions (He et al. 2016; Xue et al. 2019; Wu et al. 2024). The significantly elevated polymorphism observed within the *ycf1* and *rpl32* genes positions these genomic regions as strong candidates for use in future phylogenetic and population genetics studies. Our *K*_a_/*K*_s_ analysis revealed that most chloroplast genes undergo purifying selection, with an average *K*_a_/*K*_s_ ratio of 0.23 (Figure 6), emphasizing their functional constraints (Wicke et al. 2011; Daniell et al. 2016). However, the identification of *psbJ* in *E. longifolia* and *rpl20* in *L. floridana* showing ratios above 1 suggests positive selection in these specific lineages, warranting further investigation of these genes' role in adaptive responses (Xu et al. 2015, 2020).

Our phylogenetic analysis based on 77 protein‐coding genes, with strong statistical support (bootstrap values > 90%) (Figure 7), confirmed the monophyly of Simaroubaceae and placed *P. quassioides* firmly within this clade. The observed sister‐group relationship between Simaroubaceae and Rutaceae further resolved the Sapindales order and confirmed previous findings (Saina et al. 2018; Almeida‐Silva et al. 2024). These phylogenetic findings are particularly significant, resolving past taxonomic debates concerning the group's relationships and providing a robust evolutionary framework for subsequent studies. Ultimately, this study provides a foundational understanding of the *P. quassioides* chloroplast genome, offering a valuable genetic resource for the development of conservation strategies for this medicinal species.

## 5 Conclusions

This comprehensive analysis of the *P. quassioides* chloroplast genome reveals both conserved architectural features and dynamic evolutionary patterns within Simaroubaceae. Our findings establish patterns of molecular evolution through identified variable regions and selective pressures, while confirming the family's monophyly and its evolutionary relationship with Rutaceae. These genomic insights provide practical tools for species authentication and breeding programs, while creating a foundation for investigating the genetic basis of medicinal properties in *P. quassioides* . This research advances both our understanding of chloroplast genome evolution and the development of improved therapeutic applications from this valuable medicinal species.

## Author Contributions

**Qin Liu:** conceptualization (equal), data curation (equal), formal analysis (equal), investigation (equal), methodology (equal), writing – original draft (equal). **Huaxi Huang:** conceptualization (equal), data curation (equal), formal analysis (equal), investigation (equal), methodology (equal), writing – original draft (equal). **Jinhang Lin:** formal analysis (equal), software (equal). **Fanglin Liu:** investigation (equal), software (equal), visualization (equal). **Xuexue Wang:** validation (equal), visualization (equal). **Rong Chen:** conceptualization (equal), project administration (equal), resources (equal), supervision (equal), writing – review and editing (equal). **Xiaoshan Geng:** conceptualization (equal), project administration (equal), resources (equal), supervision (equal), writing – review and editing (equal).

## Conflicts of Interest

The authors declare no conflicts of interest.

## Data Availability

The genome sequence data that support the findings of this study are openly available in GenBank of NCBI at https://www.ncbi.nlm.nih.gov under the accession no. NC_067857.1. The associated BioProject, SRA, and Bio‐Sample numbers are PRJNA786878, SRP349634, and SAMN23730768, respectively. Analysis scripts are accessible at https://github.com/songtaste1230/Pquassioides_cpGenome_Analysis.
